# Analysis of errors made on *in utero* MR studies of the foetal brain in the MERIDIAN study

**DOI:** 10.1007/s00330-018-5508-x

**Published:** 2018-06-15

**Authors:** Ruth Batty, Mary L. Gawne-Cain, Cara Mooney, Laura Mandefield, Michael Bradburn, Gerald Mason, Paul D. Griffiths

**Affiliations:** 1 Department of Radiology, Sheffield Teaching Hospitals Trust, Sheffield, UK; 2 Department of Radiology, University Hospital Southampton NHS Foundation Trust, Sheffield, UK; 3 Clinical Trials Research Unit, School of Health and Related Research, University of Sheffield, Sheffield, UK; 4 Fetal Medicine Unit, Leeds Teaching Hospitals NHS Trust, Leeds, UK; 5 Academic Unit of Radiology, University of Sheffield, Floor C, Royal Hallamshire Hospital, Glossop Road, Sheffield, S10 2JF England

**Keywords:** Magnetic resonance, Diagnostic errors, Radiologists, Brain, Foetus

## Abstract

**Objectives:**

*In utero* magnetic resonance (iuMR) imaging to diagnose foetal brain abnormalities has been established and is supported by meta-analyses of retrospective and prospective studies. In this paper we describe and classify the iuMR errors made in the largest diagnostic accuracy study to date (MERIDIAN). We also correlate the error rates and types with the prior experience of the reporting radiologists in order to inform how to provide a national programme with the best diagnostic accuracy achievable.

**Methods:**

The MERIDIAN cohort of 570 foetus formed the basis of this study and included 40 cases with a confirmed diagnostic error, compared with the Outcome Reference Diagnosis. Analysis included the potential clinical effect of the error and classification of error type through an Expert Neuroradiological Panel re-reporting the study. Assessments were made regarding radiologists experience prior to MERIDIAN.

**Results:**

The overall confirmed error rate for iuMR was 7·0% and it was considered that there would have been an adverse effect on prognostic information in 22/40 cases if the iuMR had informed counselling. The experienced central reporter made statistically significant fewer errors than the less experienced non-central reporters (3·8% v 11·0%) and the central reporter made fewer clinically significant errors. Furthermore, the type of cognitive errors differed between central and non-central reporters.

**Conclusions:**

Although iuMR imaging improves the diagnostic accuracy of detecting foetal brain abnormalities there remains a substantial error rate, which can have major clinical significance. We have shown that error rates are lower for more experienced reporting radiologists with fewer potential deleterious clinical implications. We discuss the implications of these findings in terms of providing a uniform national service.

**Key Points:**

*• Overall confirmed error rate for iuMR diagnosing foetal brain abnormalities was 7·0%.*

*• IuMR reports had an adverse effect on counselling in 55% of error cases.*

*• Error rates are consistently lower for more experienced radiologists.*

*• Collaboration between radiologists, dual reporting, overseeing scan and formal training can reduce errors.*

**Electronic supplementary material:**

The online version of this article (10.1007/s00330-018-5508-x) contains supplementary material, which is available to authorized users.

## Introduction

The ‘Magnetic resonance imaging to enhance the diagnosis of foetal developmental brain abnormalities *in utero*’ (MERIDIAN) study was a large, prospective multicentre study performed in the UK [1]. MERIDIAN was designed to assess the diagnostic impact of performing *in utero* MR (iuMR) imaging of the foetus after antenatal ultrasonography recognised a brain abnormality. The primary and secondary outcome measures have been reported and confirm major benefits in performing iuMR imaging with a 25% increase in diagnostic accuracy and positive effects on clinical management in a high proportion of cases [1, 2].

Further analyses of the MERIDIAN data have been performed based on suggestions from an expert clinical advisers group who contributed at the concept stage of the study in the form of three anatomical sub-group studies [3–5]. A further suggestion was to undertake a detailed review of cases in which the iuMR reports were incorrect, which we report in this paper along with classification of the types of errors made. We correlate the errors with the previous experience of the radiologist in order to draw conclusions about future training programmes and planning iuMR services in the UK.

## Materials and methods

### Ethics and participants

Ethics approval was obtained for a multicentre study through the Integrated Research Application System (62734). Details of recruitment, consent and numbers of participants are presented elsewhere [1] but the main report concerned 570 foetus whose mothers provided written informed consent, had the iuMR imaging study within 2 weeks of the ultrasound and Outcome Reference Diagnosis (ORD) was available. In 41/570 cases the diagnosis from iuMR imaging was recorded as being incorrect in comparison with 183/570 incorrect diagnoses on ultrasound.

### Clinical significance of iuMR errors

A retrospective evaluation of the potential clinical significance of the iuMR error was made, based on the original iuMR report and the ORD only. The iuMR and ORD reports were reviewed by an experienced foetal maternal expert (GM) who judged if the discrepancies were likely to have led to changes in discussions about prognosis and/or termination of pregnancy (TOP) using the following classification (modified from Taylor et al. [6]).

A. The disagreement in diagnoses was not likely to have changed discussions about prognosis
B. The disagreement in diagnoses was likely to have changed discussions about prognosis, but not about TOP
C. The disagreement in diagnoses was likely to have changed discussions concerning TOP.

The assessor was not aware of scanning centre/radiologist that performed and reported the iuMR examination.

### The expert neuroradiology panel (ENP)

The 41 iuMR errors were consensus-reported by an ENP consisting of two neuroradiologists with sub-speciality interest and experience in paediatric/foetal neuroimaging (RB and MGC). The ENP had the same information that was available to the original reporter, namely the free text ultrasound report and tabulated pathology diagnoses with the certainty of each diagnosis but were blinded to which scanning centre/radiologist performed and reported the study. The ENP reported the iuMR study in the same manner as in MERIDIAN using comparable paperwork and commented on each diagnosis made on ultrasound, using ‘diagnosis excluded’ for disagreements and adding further diagnoses where appropriate. After making their report, the ENP read the ORD and re-reviewed the case answering the following questions:

Does your report agree with the ORD? If not-

- Does the ENP now agree with the ORD? (i.e. after review the ENP’s original diagnosis was considered incorrect) or
- Does the ENP maintain their original diagnosis was correct (i.e. the ENP suspects that ORD is incorrect).

The ENP was then given the original iuMR report from the MERIDIAN study and having compared it with their own report, placed each case into one of three groups:

- Group 1 – ENP report agrees with the ORD and not the original iuMR report
- Group 2 – ENP agrees with the original iuMR report (not with the ORD)
- Group 3 – there were discrepancies between all three reports (original iuMR, ORD and ENP iuMR report).

Where the ENP disagreed with the original iuMR (groups 1 and 3) they were asked to predict the type of cognitive error that best explained the mistake from the following options (modified from references [6, 7]):

- **Perceptual error** – The diagnostic finding is recognisable but missed by the radiologist leading to under-reporting of pathology. It is predicted that the radiologist would have reported this correctly on another occasion.
- **Inadequate knowledge base** – Insufficient knowledge of the relevant condition or the gestational age-dependent normal appearances at the time of reporting. It is predicted that the radiologist would repeat the mistake without further training and/or experience.
- **Faulty or incomplete test performance** – The radiologist has not appreciated that the poor quality of the images has limited the chance of arriving at the correct diagnosis. If supervising the iuMR study the radiologist should have requested repeat/further sequences or recalled the woman for further imaging.
- **Over-interpretation of findings** – importance and/or relevance of observations are over-emphasised
- **Premature closure** – Failure to consider other diagnostic possibilities once an initial diagnosis has been reached, e.g. ‘one abnormality correctly reported, forgot to look for associated abnormalities’.
- **Faulty interpretation** – diagnostic finding is correctly identified but an incorrect conclusion is made.

### Experience of the reporting radiologist

The experience of each radiologist was categorised by how many foetal brain iuMR studies they had reported prior to MERIDIAN. Approximately two-thirds of the iuMR imaging studies were performed at the central site (Sheffield, UK) and were reported by the Chief Investigator (PDG – ‘central reporter’) who had the most previous experience (> 1,000 cases). The other studies were performed at one of five regional centres and reported by local radiologists (‘non-central reporters’). At four of the sites the iuMR studies were performed by one local radiologist, whilst at the other they were reported by a number of radiologists. Previous experience of the non-central reporters was grouped into; ≤ 50 cases, 51–150 cases and 151–300 cases. The overall error rate was calculated for central and non-central reporters and the difference analysed using a chi-squared test. Overall error rate and error rate by number of cases reported in the study (first 25, 26–50, 51–75 and 75+) was calculated for each previous experience category.

## Results

Forty-one iuMR errors were reported among the 570 foetus in the MERIDIAN primary cohort (7·2% error rate). The ENP discovered one case where a transcriptional mistake had been made and the iuMR (and ultrasound) agreed with ORD, leaving 40 genuine errors (7·0%). The incidence of errors was similar in relation to gestational age with 28 errors in foetuses aged 18–23 gestational weeks (error rate 7·6%) and 12 among foetuses aged ≥ 24 weeks (error rate 6·0%). In 21/40 cases iuMR and ultrasound made the same, incorrect diagnosis, whilst in 17/40 iuMR and ultrasound made different, incorrect diagnoses. In 2/40 cases ultrasound was correct but iuMR incorrect.

### Clinical significance of iuMR errors

When compared with ORD, incorrect iuMR reports were considered to have had an adverse effect on discussions concerning prognosis and/or TOP in 22/40 (55%) cases (15 category B and seven category C). The central reporter was responsible for 12 errors (two category B and two category C). The non-central reporters were responsible for 28 errors (13 category B and five category C). These features are summarised in Table 1 and detailed clinical descriptions are provided in the Electronic Supplementary Material (ESM), Tables 1 and 2.

**Table 1:** The potential clinical implications of the iuMR errors made by the central and non-central reporters as judged by a Foetal Medicine expert. Implication A means ‘The disagreement in diagnoses was not likely to have changed discussions about prognosis’, implication B – ‘The disagreement in diagnoses was likely to have changed discussions about prognosis, but not about TOP’ and implication C – ‘The disagreement in diagnoses was likely to have changed discussions concerning TOP’. The confidence of the incorrect diagnoses is also shown (High = 70% or 90% certainty, Low = 10%, 30% or 50% certainty)

	N (%)	Potential clinical relevance			Confidence level of diagnosis	
		A	B	C	High	Low
Central	12/316 (3·8%)	8	2	2	11	1
Non-central	28/254 (11·0%)	10	13	5	20	8

### Analysis by the ENP

**Fig. 1:**
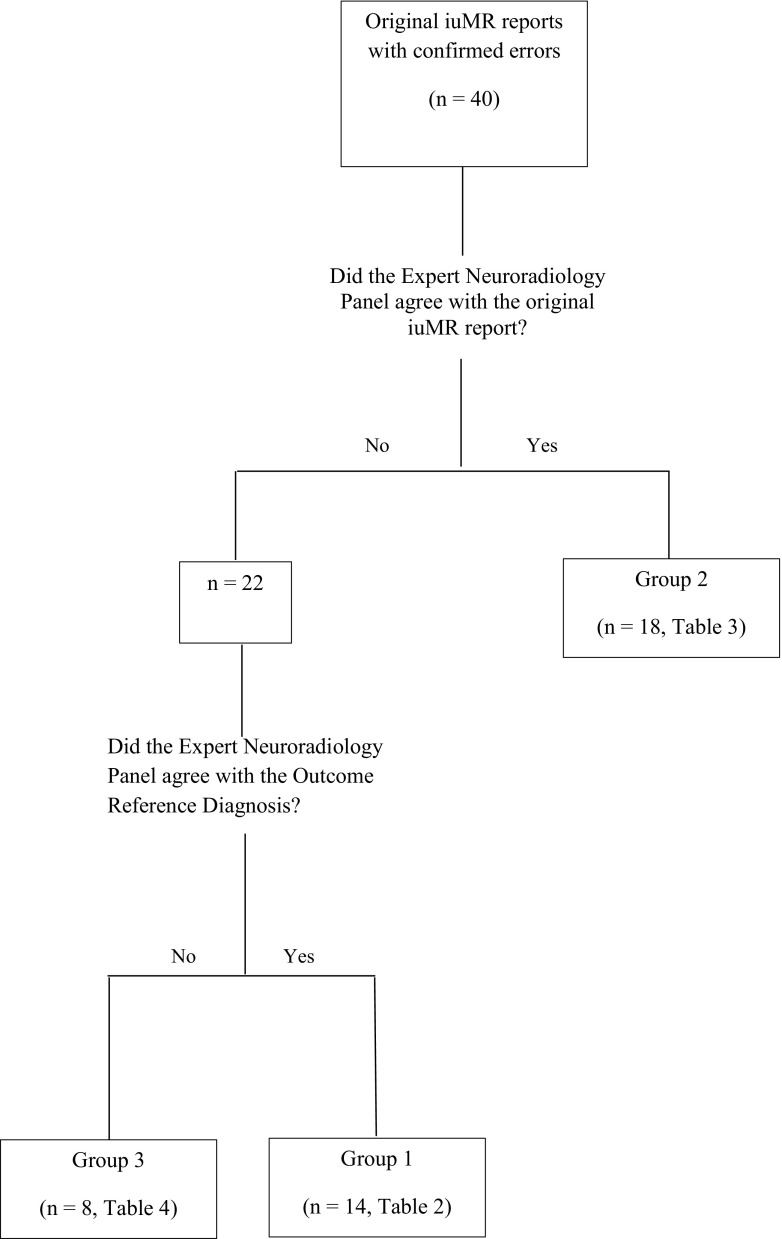
A flowchart showing the classification of errors made on iuMR imaging in relation to the opinion of the Expert Neuroradiology Panel used for analysis in this paper

The ENP reports are summarised in Fig. 1 and detailed in ESM Tables 3, 4 and 5.

#### Group 1

Fourteen of 40 cases (35%) of errors were classified as Group 1. The central reporter contributed to 1/14 (7%) of those cases, the other 13/14 (93%) were from the non-central reporters. The most common anatomical areas for error were: corpus callosum (4/14 cases), posterior fossa abnormalities (3/14 cases) and sulcation/gyration abnormalities (2/14). The type of cognitive errors made on the original iuMR reports were judged to be: inadequate knowledge base (5), over-interpretation of findings (4), faulty or incomplete test performance (3) and perceptual error (2).

#### Group 2

Eighteen of 40 cases (45%) were classified as Group 2 errors. The central reporter made 10/18 (56%) of these errors and the other 8/18 (44%) resulted from non-central reporters (Table 4). In 17/18 cases the ENP maintained that the original iuMR report was correct even after ORD was revealed. In one case the ENP changed their opinion in light of the ORD; specifically they recognised that lobar holoprosencephaly had been overlooked on their first analysis and the original iuMR report. Cognitive error analysis was not appropriate in this group because ENP agreed with the original MR reports.

#### Group 3

Eight of 40 (20%) cases were classified as Group 3. The central reporter contributed to 1/8 (12·5%) of those cases, the other 7/8 (87·5%) resulting from non-central reporters. The cognitive error causing the mistake was judged to be: inadequate knowledge base (1), over-interpretation of findings (2), faulty or incomplete test performance (3) and perceptual error (1). In the remaining case when all of the information was considered, ENP judged that the original iuMR was correct and no cognitive error could be attributed.

### Experience of the reporting radiologist

The central reporter had the most amount of previous experience of iuMR brain studies (> 1,000) and produced 12/40 (30%) iuMR errors after reporting 316/570 (55%) of the cohort, giving an overall error rate 3·8%. The non-central reporters produced 28/40 (70%) iuMR errors and reported 254/570 (45%) of the cohort, giving a combined error rate of 11·0% (Table 2). The margin of 7·2% is statistically significantly different (95% CI: 2·5–12·0, *p*=0·001). The ENP agreed with the original iuMR report, and not with ORD, in 10/12 (83%) of the cases reported by the central reporter and in 8/28 (29%) of non-central reports.

**Table 2:** The distribution of the errors made by the central and non-central reporters in relation to the Group classification made by the Expert Neuroradiology Panel

	Group 1 errors ENP agrees with ORD (n=14)	Group 2 errors ENP agrees with original iuMR report (n=18)	Group 3 errors ENP, ORD and original iuMR report discrepant (n=8)	Overall error rate
Central reporter	1/12 (8%)	10/12 (84%)	1/12 (8%)	12/316 (3·8%)
Non-central reporters	13/28 (46%)	8/28 (29%)	7/28 (25%)	28/254 (11·0%)
				Difference and 95% CI: 7·2 (2·5-12·0%) *p*-value*=0·001

*Differences analysed by the Chi-squared test

The error rate of the central reporter was consistently less than 5% (Table 3). Although the numbers are limited, there appears to be an association between error rate and level of previous experience, with the lower experienced radiologists having higher error rates of greater than 10%. Non-central, experienced radiologists have error rates of less than 10%, although the total number of scans this is based on is low (n=71). In addition, although it is difficult to comment on the change in error rate during the study as the radiologists reported a varying number of cases, there does appear to be a trend towards improving accuracy for the non-central reporters during the course of MERIDIAN.

**Table 3:** Error rates in relation to the experience category of the reporting radiologist. See text for details

Experience category (number of radiologists)	Number of cases reported in MERIDIAN: Overall error rate	Error rate in the radiologists’ first 25 reports	Error rate in the radiologists’ reports 26–50	Error rate in the radiologists’ reports 51–75	Error rate in the radiologists’ reports 76+
Non central reporter 0–50 iuMR studies (3)	95: 11·6%	13·0% (of 54 cases)	16·0% (of 25 cases)	0·0% (of 16 cases)	Not performed
Non central reporter 50–150 iuMR studies (4)	138: 12·3%	13·8% (of 87 cases)	10·8% (of 37 cases)	7·1% (of 14 cases)	Not performed
Non central reporter 150–300 iuMR studies (4)	71: 8·5%	9·4% (of 64 cases)	0·0% (of 7 cases)	Not performed	Not performed
Central reporter >1,000 iuMR studies (1)	316: 3·8%	4·0% (of 25 cases)	0·0% (of 25 cases)	0·0% (of 25 cases)	4·6% (of 241 cases)

The ENP made assessments of the likely cognitive errors for Groups 1 and 3 cases, the ENP changed their opinion on one Group 3 case to agree with the original iuMR report after re-review, therefore 21 cognitive errors were commented on. The central reporter was responsible for 2/21 and in both cases the error was ‘Over-interpretation of findings’. The non-central reporters were responsible for 19/21 errors with a cognitive cause, consisting of ‘Incomplete test performance – poor quality images’ (6), ‘Inadequate knowledge base’ (5), ‘Over-interpretation of findings’ (5) and ‘Perceptual error’ (3).

## Discussion

The error rate from the iuMR imaging reports in the MERIDIAN study was 7·0% (40 cases) and in this paper we have reported an analysis of those errors. The incorrect iuMR reports were expected to change in counselling with or without changed discussions concerning termination of pregnancy in 22/40 (55% – category B and C). It should be appreciated, however, that in 38/40 (95%) of cases the ultrasound report was also incorrect. Experience appears to be relevant as the error rate for the central, and most experienced, reporter was approximately one-third that of the less experienced, non-central reporters (3·8% vs. 11·0%), a result that reached statistical significance. In addition, the errors made by the central reporter were less likely to have affected discussions concerning prognosis or termination of pregnancy when compared with the non-central reports (33% vs. 64%).

We produced three ‘error’ groups when the expert report was compared with the original iuMR and the ORD. Group 1 errors occurred when the ENP agreed with the ORD, the implication being the error on the original iuMR report would not have been made by other neuroradiologists and probably reflects a ‘genuine radiological’ mistake (35% of errors). Group 2 errors occurred when the ENP agreed with the original iuMR report (45% of errors). The two most likely explanations are (i) the pathology was too subtle to be diagnosed and all neuroradiologists would make the same mistake or (ii) the ORD is incorrect. It seems counter-intuitive to question the veracity of ORD, but it should be appreciated that an ORD may change with time, at a point when more information is available [8]. The ORD for MERIDIAN was either autopsy or postnatal imaging up to 6 months of age, often by cranial ultrasonography, which is not sensitive for subtle intracranial pathology. It also has specific problems in visualising the contents of the posterior fossa and 50% of Group 2 errors involved some disagreement regarding the posterior fossa. The different ORD methods (autopsy or post-natal imaging including post-natal MR, post-natal CT and cranial ultrasonography) may be a limitation to the study as the diagnostic accuracy of these methods vary. The follow-on studies to MERIDIAN will refresh ORD as it becomes available and cases will be scrutinised again with improved reference standards [9]. Group 3 (20% of errors) were cases in which the original iuMR report, ENP report and ORD were at variance and one interpretation is that they were complex cases and reliable prenatal diagnosis was impossible. There were differences in the categories of error made in relation to the experience of the reporters. Eighty-three percent of the errors made by the central reporter were Group 2 (ENP agreed with original report), whereas the most common category of error for the non-central reporters was Group 1 (46% – probably representing ‘genuine’ radiological errors).

Further evidence to support the importance of experience comes from studying error rates based on self-reported previous experience of the radiologist. Every radiologist, irrespective of experience, had an improvement in diagnostic accuracy over ultrasound of greater than 10%, but there were variations. Radiologists with more previous experience were less likely to make errors, those who had reported 150–300 previous scans consistently showed a less than 10% error rate and the central reporter was consistently under 5%. In the two lower experience categories, errors appeared to occur early on although it is difficult to generalise due to varying numbers of total scans.

The investigation of the causes of error in diagnostic imaging is important because of reported high rates and adverse clinical consequences [6, 7]. This is likely to be the case in diagnosis of foetal brain abnormalities as TOP is the only intervention available. Ideally the investigation should involve questioning the person who made the error close to the time the mistake was made. This is generally difficult to do, but was impossible here because the time between reporting the iuMR study and the error being highlighted on ORD was over 6 months. This delay may also have had an impact on the diagnostic accuracy of ORD in the developing brain. We had to rely on retrospective assessments by the ENP and we are aware of the inherent weaknesses of this approach. A recent review by Lee et al. states that most diagnostic errors can be attributed to ‘Cognitive’ or ‘System-related’ errors, or a combination of them [7]. Those authors cite examples from both sources and propose corrective strategies to mitigate the risks. System-related errors arise from technical and equipment failures, or poor communication, policies or procedures. It was considered impossible to review system-related errors in this study and we have concentrated on cognitive errors.

Cognitive errors are thought to be the most common type of error in internal medicine, occurring in approximately three-quarters of mistakes and arise from problems with perception, heuristics (‘mental shortcuts’ arising from pattern recognition) and biases, alone or in combination [7, 10]. Taylor et al. used that framework to analyse diagnostic errors in paediatric radiology and refined the nature of cognitive errors occurring in radiological practice [6]. We found only one paper that has attempted to analyse reports from iuMR studies, specifically from 200 cases of foetal ventriculomegaly [11]. That study, however, did not have access to outcome reference data and as a result the authors could comment only on the variability in the reports between groups of radiologists from different specialties, which they found to be wide. We have used a modified version of Taylor’s approach using six domains customised for analysis of iuMR studies. However, the ENP only attributed errors into four of those categories – ‘perceptual errors’, ‘incomplete test performance’, ‘inadequate knowledge base’ and ‘over-interpretation of findings’. The ENP proposed the type of cognitive error in 21 cases (14/14 Group 1 and 7/8 Group 3). All of the errors resulting from faulty or incomplete test performance (poor image quality) occurred at non-central sites, which is relevant because at the central site all iuMR studies were overseen by the central reporter, which was not routinely the case at non-central sites. The major advantages of the reporting radiologist overseeing the procedure relates to either stopping the test early if all of the information has been obtained or prolonging the study to ensure images are of diagnostic quality.

In summary, iuMR imaging improves the diagnostic accuracy of detecting foetal brain abnormalities but there is still a substantial error rate. We have shown differences in error rates that appear to be related to the experience of the radiologist reporter and that the type of error and potential clinical implications also correlate with experience. This has substantial implications for planning a service that includes iuMR imaging because it is essential that a radiologist has sufficient training and exposure to cases to gain and maintain competence. Our estimate of the prevalence of brain abnormalities in foetuses ≥ 18 gw in the MERIDIAN study is 1/1,000 pregnancies and it is a major challenge to see sufficient number of cases at most single centres in the UK to attain and maintain competency. There are several approaches to this issue including providing iuMR studies only at a small number of supra-regional centres and/or promoting collaboration between radiologists to form expert panels for cases. Strategies can also be put in place to mitigate the risk of making errors, e.g. formal training should reduce errors arising from ‘inadequate knowledge base’, supervision of the iuMR study by the radiologist may reduce errors from ‘faulty or incomplete test performance’ and double reporting of images should reduce ‘perceptual errors’ and increase exposure. These strategies will increase the workload of radiologists substantially.

## Electronic supplementary material

ESM 1 (DOCX 34 kb)

## Abbreviations

ENP: The Expert Neuroradiology Panel
iuMR: In utero magnetic resonance imaging
ORD: Outcome Reference Diagnosis
TOP: Termination of pregnancy
