# Supplementary material for: Analysis of errors made on in utero MR studies of the foetal brain in the MERIDIAN study
Source: Eur Radiol. 2018 Jun 15;29(1):195–201. doi: 10.1007/s00330-018-5508-x (PMC6291426; doi:10.1007/s00330-018-5508-x)
Supplement: Supplementary file 1 — (DOCX 34 kb) [file 330_2018_5508_MOESM1_ESM.docx]

**ELECTRONIC SUPPLEMENTARY MATERIAL**

**ESM Table 1.** Imaging summaries of and potential clinical significance of iuMR imaging errors made by the central reporter. Implication A means ‘The disagreement in diagnoses was not likely to have changed discussions about prognosis’, implication B – ‘The disagreement in diagnoses was likely to have changed discussions about prognosis, but not about TOP’ and implication C – ‘The disagreement in diagnoses was likely to have changed discussions concerning TOP’.

| **GW at iuMR** | **Original iuMR findings (confidence level)** | **ORD**  **Type:findings** | **Potential**  **clinical**  **relevance** |
| --- | --- | --- | --- |
| **23w** | VM (high) | PNMR:  Hypogenesis of CC | B |
| **20w** | VM (high)  Chiari 2 malformation (high)  Heterotopia (high) | PNMR: Lobar holoprosencephaly  Heterotopia | A |
| **22w** | Enlarged CM (high) | PNUSS: Normal | A |
| **26w** | Dandy Walker  Malformation (low) | Autopsy: Dandy Walker  Malformation  Agenesis CC | C |
| **35w** | VM (high)  Encephalomalacia (high) | PNMR: VM,  Encephalomalacia Septo-optic dysplasia | A |
| **34w** | VM (high),  Hypogenesis CC (high)  Polymicrogyria (low) | PNMR: Hypogenesis CC | C |
| **31w** | Microcephaly (high) | PNUSS: Normal | A |
| **22w** | Interhemispheric cyst (low) | PNUSS: Normal | A |
| **37w** | Enlarged CM (high) | PNUSS: Normal | A |
| **19w** | VM (high)  Hypogenesis CC (high) | PNMR: VM | B |
| **21w** | Posterior fossa arachnoid cyst (high) | PNUSS: Normal | A |
| **22w** | Enlarged CM (high) | PNUSS: Normal | A |

**ESM Table 2.** Imaging summaries of and potential clinical significance of iuMR imaging errors made by the non-central reporter. Implication A means ‘The disagreement in diagnoses was not likely to have changed discussions about prognosis’, implication B – ‘The disagreement in diagnoses was likely to have changed discussions about prognosis, but not about TOP’ and implication C – ‘The disagreement in diagnoses was likely to have changed discussions concerning TOP’.

| **GW at iuMR** | **Original iuMR findings (confidence level)** | **ORD**  **Type:findings** | **Potential**  **clinical**  **relevance** |
| --- | --- | --- | --- |
| **21w** | Cerebellar hypogenesis (high) | PNMR: Posterior fossa arachnoid cyst | A |
| **34w** | VM (high)  Absent CSP | PNMR: VM  Septo-optic dysplasia  Enlarged CM | B |
| **29w** | VM (high)  Hypogenesis vermis, (low)  Enlarged CM (High) | PNUSS: VM  Enlarged CM | A |
| **22w** | VM (low) | PNMR: VM  Heterotopia | C |
| **20w** | Inter-hemispheric cyst (high)  Hypogenesis CC (high), | PNMR: Intra-ventricular mass lesion | C |
| **20w** | Encephalocoele (high),  Dandy Walker Spectrum (high) | Autopsy: Encephalocoele | A |
| **21w** | Periventricular leukomalacia (low) | Autopsy: Normal brain. (Beckwith-Wiedemann  Syndrome) | B |
| **21w** | Hypogenesis vermis (low) | PNMR: Normal | A |
| **23w** | VM (high) | PNUSS: VM,  Hypogenesis CC | B |
| **20w** | VM (high) | PNMR: VM  Hypogenesis CC | B |
| **21w** | VM,  Hypogenesis CC (low)  Focal encephalomalacia (low) | Autopsy: VM | B |
| **23w** | Agenesis CC (high) | Autopsy: Agenesis CC, Heterotopia | B |
| **29w** | Frontal cysts (low)  Focal encephalomalacia (high) | PNUSS: normal brain | C |
| **21w** | Enlarged CM (high) | PNUSS: Normal | A |
| **21w** | VM (high) | PNMR: VM  Lobar holoprosencephaly | C |
| **21w** | VM (high)  Sulcation delay (low) | PNMR: Germinal matrix haemorrhage | A |
| **20w** | Hypoplasia vermis (low) | PNMR: Normal | A |
| **32w** | Germinal matrix haemorrhage (high) | PNUSS: Normal | B |
| **24w** | VM (high)  Agenesis CC (high) | PNMR: Agenesis CC  Schizencephaly | B |
| **25w** | VM (high)  Sulcation delay (low) Microcephaly (high) | PNUSS: Microcephaly | A |
| **23w** | VM (high)  Sulcation delay (high) | PNMR: VM  Hypogenesis CC and  Inter-hemispheric cyst | B |
| **22w** | VM (high) | PNMR: VM  Hypogenesis CC | B |
| **22w** | VM (high)  Agenesis CC (high) | PNMR: VM  Lobar holoprosencephaly  Heterotopia | B |
| **22w** | VM (high)  Hypogenesis CC with cyst (high) | Autopsy: Supra-sellar vascular malformation | B |
| **20w** | VM (high)  Agenesis CC (high) | PNMR: VM | C |
| **22w** | Hypogenesis vermis (high) | PNUSS: Normal | A |
| **21w** | VM (high) | Autopsy: VM  Chiari 2 malformation | A (because the spinal abnormality was known) |
| **24w** | Hypogenesis CC (high) | PNUSS: Normal | B |

**ESM Table 3.** Clinical and imaging summaries of cases classified as Group 1 errors by the Expert Neuroradiology Panel (ENP agreed with the Outcome Reference Diagnosis and disagreed with the original iuMR report). *Abbreviations: CC – corpus callosum, CM – cisterna magna, CSF – cerebro-spinal fluid, CSP – cavum septum pellucidum, PNMR – postnatal MR imaging, PNUSS – postnatal cranial ultrasonography, VM – ventriculomegaly, gw – gestational weeks.*

| **GW at iuMR** | **ORD Type:report which agreed with ENP** | **Diagnosis on original iuMR report** | **Error type on original MR report** |
| --- | --- | --- | --- |
| 34gw | PNMR: VM  Absent CSP  Enlarged CM | VM  Absent CSP | Perceptual error,  (CM not measured) |
| 21gw | Autopsy: normal brain | Periventricular leukomalacia | Incomplete test performance  Poor quality images |
| 29gw | PNUSS: Subependymal cysts | Encephalomalacia in posterior white matter | Inadequate knowledge base, Misinterpreted normal structures |
| 20gw | PNMR: Normal | Hypogenesis cerebellar vermis | Over interpretation of findings  Normal growth charts should have been used |
| 24gw | PNMR: VM  Agenesis of CC with cyst, Schizencephaly | VM  Agenesis of CC | Perceptual error,  CSF clefts present |
| 25gw | PNUSS: Microcephaly | Sulcation delay | Inadequate knowledge base, Normal sulcation |
| 23gw | PNMR: VM  Hypoplasia of CC with cyst | VM  Sulcation delay | Inadequate knowledge base, Normal sulcation |
| 22gw | PNMR: VM  hypoplasia of CC | VM | Incomplete test performance  Poor quality images |
| 20gw | PNMR: VM | VM  Agenesis of CC | Over interpretation of findings  Unknown cause |
| 22gw | PNUSS: Normal | Hypogenesis cerebellar vermis | Over interpretation of findings  Normal growth charts should have been used |
| 21gw | Autopsy: VM  Chiari 2 malformation | VM | Inadequate knowledge base,  Open spinal defect present |
| 24gw | PNUSS: VM | Hypoplasia of CC | Incomplete test performance  Poor quality images |
| 31gw | PNUSS: Normal | Microcephaly | Over interpretation of findings  Normal growth charts should have been used |
| 22gw | PNUSS: Normal | Inter-hemispheric cyst | Over interpretation of findings,  Incorrect interpretation of wide extra-axial spaces |

**ESM Table 4.** Clinical and imaging summaries of cases classified as Group 2 errors by the Expert Neuroradiology Panel (ENP agreed with the original iuMR report and disagreed with the Outcome Reference Diagnosis). *Abbreviations: CC – corpus callosum, CFA – cortical formation abnormality, CSP – cavum septum pellucidum, HPE – holoprosencephaly, PNMR – postnatal MR imaging, PNUSS – postnatal cranial ultrasonography, TCD – trans-cerebellar diameter, VM – ventriculomegaly, gw – gestational weeks.*

| **GW at iuMR** | **ENP and original iuMR reports** | **ORD**  **Type:report** | **Retrospective agreement with ORD** |
| --- | --- | --- | --- |
| 21gw | Cerebellar hypoplasia | PNMR: Posterior fossa arachnoid cyst | No, based on TCD measurement |
| 20gw | Encephalocoele  Dandy Walker Spectrum | Autopsy: Encephalocoele | No |
| 21gw | Cerebellar hypoplasia | PNMR: Normal brain | No, based on TCD measurement |
| 20gw | VM | PNMR: VM  Hypogenesis CC | No |
| 23gw | Agenesis CC | Autopsy: Agenesis CC  Heterotopia | No |
| 23gw | VM | PNMR: Hypogenesis CC | No |
| 21gw | Enlarged CM | PNUSS: Normal brain | No |
| 32gw | Germinal matrix haemorrhage | PNUSS: Normal brain | No |
| 22gw | VM  Agenesis CC | PNMR: Lobar HPE  Extensive CFA | Partial.  HPE agreed CFA not visualised |
| 22gw | Enlarged CM | PNUSS: Normal brain | No, based on CM measurement |
| 22gw | Hypogenesis CC and cyst | Autopsy: Normal CC  Supra-sellar vascular malformation | No |
| 26gw | Dandy Walker Spectrum | Autopsy: Dandy Walker Spectrum  Agenesis CC | No |
| 35gw | VM  Encephalomalacia | PNMR:VM  Encephalomalacia  Septo-optic dysplasia | No. CSP present on iuMR |
| 34gw | VM  Hypogenesis CC  Polymicrogyria | PNMR: Hypogenesis CC | No |
| 37gw | Enlarged CM | PNUSS: Normal brain | No |
| 19gw | VM  Hypogenesis CC | PNMR: VM | No |
| 21gw | Posterior fossa arachnoid cyst 2 | PNUSS: Normal brain | No |
| 22gw | Enlarged CM | PNUSS: Normal brain | No |

**ESM Table 5.** Clinical and imaging summaries of cases classified as Group 3 errors by the Expert Neuroradiology Panel (the original iuMR report, the Expert Neuroradiology Panel report and the Outcome Reference Diagnosis were all discrepant.). *Abbreviations: CC – corpus callosum, CM – cisterna magna, HPE – holoprosencephaly, PNMR – postnatal MR imaging, PNUSS – postnatal cranial ultrasonography, TCD – trans-cerebellar diameter, VM – ventriculomegaly, gw – gestational weeks.*

| **GW at iuMR** | **Diagnosis on original iuMR** | **ORD**  **Type: Report** | **Diagnosis on ENP** | **ENP opinion after ORD available** | **Analysis of error type on the original iuMR assessed by ENP** |
| --- | --- | --- | --- | --- | --- |
| 29gw | VM  Enlarged CM  Hypoplasia of vermis | PNUSS:  VM | VM  Enlarged CM | Unchanged  (CM >10mm) | Over interpretation of findings  Normal growth charts should have been used |
| 22gw | VM | PNMR:VM  Heterotopia | VM  Enlarged CM | Unchanged  (could not see heterotopia) | Incomplete test performance  Poor quality images |
| 20gw | VM  Extra-axial arachnoid cyst  Polymicrogyria | PNMR:Intra-ventricular mass lesion | VM  Extra-axial  Arachnoid cyst | Unchanged (no intra-ventricular mass seen) | Inadequate knowledge base  Normal sulcation |
| 23gw | VM | PNUSS:VM  Hypogenesis of CC | VM  Rhombencephalosynapsis | Unchanged (CC seen in full) | Incomplete test performance  Poor quality images |
| 21gw | VM  Hypogenesis of CC  Focal encephalomalacia | Autopsy:  VM | VM  Focal encephalo- malacia | Unchanged (parenchymal injury present) | Over interpretation of findings  Unknown cause |
| 21gw | VM | PNMR: VM  Lobar HPE | VM  Hypoplastic cerebellum | Unchanged  (Lobar HPE refuted, CSP seen) | Perceptual error,  Unknown cause |
| 21gw | VM  Delayed gyral pattern | PNMR:  Germinal matrix haemorrhage | VM  Cerebellar hypoplasia | Unchanged  (TCD 16mm, no abn. in germinal matrix). | Incomplete test performance  Poor quality images |
| 20gw | VM  Chiari 2 Malformation  Heterotopia | PNMR: Lobar HPE  Heterotopia | VM  Chiari 2 Malformation | Partial  Heterotopia agreed  HPE not seen  Open spinal dysraphism present. | Not applicable, ENP agreed that heterotopia was present on re-review |
